# Editorial: Endocrine and Paracrine Role of FGF23 and Klotho in Health and Disease

**DOI:** 10.3389/fendo.2019.00002

**Published:** 2019-01-21

**Authors:** Reinhold G. Erben

**Affiliations:** Department of Biomedical Sciences, University of Veterinary Medicine Vienna, Vienna, Austria

**Keywords:** Klotho, fibroblast growth factor (FCF-23), chronic kidney disease, X-linked hypophosphatemia, myocardial infarction, bone mineralization, left ventricular hypertrophy

αKlotho and fibroblast growth factor-23 (FGF23) were discovered independently about 20 years ago. The transmembrane protein αKlotho was originally described as an anti-aging factor (1), whereas FGF23 was discovered as a plasma phosphate-lowering hormone, suppressing phosphate reabsorption and vitamin D hormone production in the kidney (2–4). Typical for seminal discoveries, both findings have generated an avalanche of subsequent research. It took about 10 years after these initial, completely independent discoveries to find out that both molecules are part of the same signaling pathway (5). After 20 years of intensive research, there is still keen interest in the αKlotho/FGF23 biology field. This interest is currently mainly fueled by the so far unexplained finding that the levels of circulating FGF23 are a strong predictor of disease progression and mortality in patients with chronic kidney disease (CKD), and by the emerging role of FGF23 in cardiovascular diseases (6–8). Progress in science is often driven by controversies. The αKlotho/FGF23 field is actually a very good example of controversy-driven advancement of science.

The purpose of this Research Topic was to bring together Reviews/Mini-Reviews and Original Research articles, reflecting the state of the art and the current controversies in this rapidly expanding research area. The 11 accepted articles consist of three Original Research articles and 8 Reviews or Mini-Reviews.

The Original Research articles deal (i) with the bone-kidney-gut endocrine signaling network, (ii) with the role of FGF23 as pro-hypertrophic factor in the heart, and (iii) with the function of FGF23 as a local regulator of mineralization in bone. Fujii et al. investigated the role of osteocytes in the control of mineral homeostasis, using a diphtheria toxin-mediated osteocyte ablation model in mice. Intriguingly, they found an only transient reduction in circulating FGF23 after osteocyte ablation, which was followed by profoundly increased renal phosphate excretion and suppressed renal Klotho expression after serum FGF23 had normalized. Data from human studies have shown a strong association between left ventricular hypertrophy and circulating FGF23 (6). To elucidate further the pathophysiological role of FGF23 in the development of cardiac hypertrophy, Leifheit-Nestler et al. compared two mouse models characterized by increased circulating FGF23, Klotho deficient mice and *Hyp* mice. *Hyp* mice are a model of X-linked hypophosphatemia (XLH). Leifheit-Nestler et al. found that despite high circulating FGF23, increased cardiac abundance of *Fgf23* mRNA, and low renal Klotho expression, *Hyp* mice did not show pathological cardiac remodeling. These findings suggest that increased circulating FGF23 alone is not cardiotoxic in the absence of hyperphosphatemia/hypercalcemia. If extrapolated to humans, the data provided by this study suggest that chronically elevated FGF23 levels in XLH patients will not induce left ventricular hypertrophy *per se*. It is well-known that the mineral-bone-disorder in CKD patients (CKD-MBD) is associated with impaired bone mineralization (9). It was previously thought that the mineralization defect in CKD is mainly due to secondary hyperparathyroidism, metabolic acidosis, increased circulating Wnt inhibitors, and uremic toxins (9). The paper by Andrukhova et al. establishes a new paradigm, namely that excessive osteocytic FGF23 secretion locally contributes to the bone mineralization defect in CKD-MBD by suppression of alkaline phosphatase, which in turn leads to the accumulation of the mineralization inhibitor pyrophosphate.

The 8 Reviews/Mini-Reviews cover most of the current knowledge in the FGF23/αKlotho field. The physiological actions of FGF23 are reviewed in the paper by Erben. Current evidence suggests that under physiological conditions, kidney and bone are the main target organs of FGF23 activity. The endocrine actions of FGF23 in the kidney are mediated through the FGF receptor 1c/αKlotho receptor complex, whereas the paracrine actions of FGF23 in bone are αKlotho-independent. One of the burning questions in the field is the question whether soluble Klotho has FGF23-independent effects. It is firmly established that transmembrane αKlotho functions as a co-receptor for FGF23 signaling (5). In addition, the extracellular part of αKlotho can be shed by membrane-anchored sheddases, giving rise to soluble αKlotho circulating in the blood stream. The review by Dalton et al. summarizes the state of the art as of 2017, describing the FGF23-independent hormonal and enzymatic functions of soluble αKlotho reported previously. However, in January 2018 the paradigm-shifting paper by Chen et al. (10) demonstrated that soluble αKlotho functions as a co-receptor for FGF23 signaling, and lacks any FGF23-independent effects. The review by Richter and Faul was accepted after the landmark paper by Chen et al. (10) came out, and is an insightful and thought-provoking paper. Richter and Faul not only describe what is currently known about the actions of FGF23 in the heart, liver, leukocytes, skeletal muscle, endothelium, lung, and CNS. In addition, Richter and Faul hypothesize that the main function of soluble αKlotho may be to act as a decoy receptor for FGF23, preventing pathological FGF23 signaling, which appears to be mainly mediated through FGF receptors 3 and 4.

Emerging evidence suggests that FGF23 may have paracrine effects in the heart. Leifheit-Nestler and Haffner summarize what is known about the local secretion of FGF23 in the heart, and comprehensively discuss the putative paracrine functions of FGF23 in the development of left ventricular hypertrophy. From a different perspective, the review by Stöhr et al. describes our current knowledge about the mechanisms how cardiac disease induces FGF23 secretion and how FGF23 may impact cardiovascular disease via canonical and non-canonical effects. Furthermore, they discuss the potential for therapeutic interventions. An important aspect of cardiovascular disease is vascular calcification, which is often found in CKD patients. Although, it is generally accepted that hyperphosphatemia is the major driving force for vascular calcification in CKD patients, αKlotho deficiency may also play a role. The review by Lang et al. summarizes our knowledge about the pathophysiological mechanisms leading to vascular calcification in two hyperphosphatemia mouse models, αKlotho deficient mice and mice treated with a vitamin D overload. In addition, Lang et al. outline the possibilities for therapeutic interventions explored in these mouse models.

During recent years, it became clear that activated immune cells are able to produce FGF23, and that FGF23 also acts on immune cells. The review by Fitzpatrick et al. is dealing with the role of FGF23 in inflammation. Fitzpatrick et al. point out that it is important to distinguish between indirect effects of FGF23 on the immune system mediated through suppression of vitamin D hormone production, and direct αKlotho-independent and αKlotho-dependent effects on granulocytes and macrophages, respectively.

In February 2018, anti-FGF23 antibody therapy was approved by the European Medicines Agency for the treatment of XLH. The paper by Fukumoto is a comprehensive review of the therapeutic role of FGF23 inhibition by antibodies or synthetic inhibitors in phosphate-wasting disorders and CKD. Fukumoto concludes that while patients with phosphate-wasting diseases such as XLH may benefit from FGF23 blocking antibodies as long as the therapy is carefully monitored, current evidence is insufficient to justify the use of inhibition of FGF23 signaling in patients with CKD.

In summary, the articles in this research topic are an excellent source of information about the current knowledge in the FGF23/αKlotho field.

## Author Contributions

The author confirms being the sole contributor of this work and has approved it for publication.

### Conflict of Interest Statement

The author declares that the research was conducted in the absence of any commercial or financial relationships that could be construed as a potential conflict of interest.
